# N-MYC Downstream Regulated Gene 4 (*NDRG4*), a Frequent Downregulated Gene through DNA Hypermethylation, plays a Tumor Suppressive Role in Esophageal Adenocarcinoma

**DOI:** 10.3390/cancers12092573

**Published:** 2020-09-10

**Authors:** Longlong Cao, Tianling Hu, Heng Lu, Dunfa Peng

**Affiliations:** 1 Department of Surgery, Miller School of Medicine, Miami, FL 33136, USA; lxc1122@miami.edu (L.C.); txh488@miami.edu (T.H.); heng.lu@med.miami.edu (H.L.); 2 Sylvester Comprehensive Cancer Center, University of Miami, Miami, FL 33136, USA

**Keywords:** NDRG4, esophageal adenocarcinoma, DNA methylation, tumor suppressor

## Abstract

**Simple Summary:**

Esophageal adenocarcinoma has become a major clinical challenge in the western world due to its rapid increasing incidence and poor overall prognosis. Understanding the molecular events of its tumorigenesis is the key to better diagnosis and development of better therapeutic strategies. In the current study we aimed to identify epigenetic alteration targets in esophageal adenocarcinoma. We focused on a candidate gene, *NDRG4* (N-myc downregulated gene 4). We found that *NDRG4* was frequent downregulated in esophageal adenocarcinoma through DNA hypermethylation of its promoter region. Re-expression of NRDG4 in cancer cells significantly suppressed tumor growth via inhibition of cell proliferation. These results will improve our understanding on how dysfunction of *NDRG4* contributes to esophageal adenocarcinoma. DNA hypermethylation of *NDRG4* may be a useful biomarker in clinical monitoring of esophageal adenocarcinoma patients.

**Abstract:**

The incidence of esophageal adenocarcinoma (EAC) has been rising dramatically in the past few decades in the United States and Western world. The N-myc downregulated gene 4 (*NDRG4*) belongs to the human NDRG family. In this study, we aimed to identify the expression levels, regulation, and functions of *NDRG4* in EAC. Using an integrative epigenetic approach, we identified genes showing significant downregulation in EAC and displaying upregulation after 5-Aza-deoxycitidine. Among these genes, likely to be regulated by DNA methylation, *NDRG4* was among the top 10 candidate genes. Analyses of TCGA (The Cancer Genome Atlas) and GEO (Gene Expression Omnibus) data sets and EAC tissue samples demonstrated that *NDRG4* was significantly downregulated in EAC (*p* < 0.05). Using Pyrosequencing technology for quantification of DNA methylation, we detected that *NDRG4* promoter methylation level was significantly higher in EAC tissue samples, as compared to normal esophagus samples (*p* < 0.01). A strong inverse correlation between *NDRG4* methylation and its gene expression levels (*r* = −0.4, *p* < 0.01) was observed. Treatment with 5-Aza restored the *NDRG4* expression, confirming that hypermethylation is a driving force for *NDRG4* silencing in EAC. Pathway and gene set enrichment analyses of TCGA data suggested that *NDRG4* is strongly associated with genes related to cell cycle regulation. Western blotting analysis showed significant downregulation of Cyclin D1, CDK4 and CDK6 in EAC cells after overexpression of NDRG4. Functionally, we found that the reconstitution of NDRG4 resulted in a significant reduction in tumor cell growth in two-dimensional (2D) and three-dimensional (3D) organotypic culture models and inhibited tumor cell proliferation as indicated by the EdU (5-ethynyl-2′-deoxyuridine) proliferation assay.

## 1. Introduction

The incidence of esophageal adenocarcinoma (EAC) has increased rapidly in the United States and Western countries over the past decades [1,2,3,4,5]. The five-year survival rate has been less than 20% due to presentation with late stage malignancies at diagnosis and lack of effective treatments [1,6]. EAC is regarded to originate from its precancerous Barrett’s esophagus (BE), in which the original esophageal squamous epithelium is replaced with metastatic columnar epithelium, through low grade dysplasia, high grade dysplasia to adenocarcinoma sequence [1,5,7].

DNA methylation is a major epigenomic modification that is associated with gene regulation [8,9,10]. Hypermethylation of gene promoter regions is associated with gene silencing of many tumor suppressor genes, such as *p16*, *CDH1* and many others [11,12,13]. We and others have reported frequent silencing of several genes, such as *GSTM2*, *GSTM3*, *GPX3*, *GPX7* and *MT3*, through aberrant DNA methylation in esophageal adenocarcinoma [14,15,16,17,18]. These epigenetic changes may wipe out protective mechanisms in Barrett’s esophagus, contributing to Barrett’s tumorigenesis [19].

The N-myc downstream regulated gene (NDRG) protein family consists of four members, *NDRG1*, *NDRG2*, *NDRG3* and *NDRG4*, which share 57–65% amino acids [20], among which *NDRG1* and *NDRG2* are the two most studied members [21,22,23]. Both *NDRG1* and *NDRG2* are downregulated in a variety of human cancers. Loss of their expression is associated with tumor invasion, angiogenesis and metastasis [21,24,25]. *NDRG4* is the last identified human NDRG member. The role of NDRG4 in human tumorigenesis is barely known and quite controversial [26,27,28,29,30]. Most of the recent studies reported an elevation of NDRG4 in brain tumors [28], where it promoted cell cycle progression and survival of glioblastoma cells. On the other hand, NDRG4 was downregulated in gastrointestinal tract cancers and acted as a potential tumor suppressor [27,29]. DNA hypermethylation of *NDRG4* was frequently detected in colorectal cancers [27]. *NDRG4* was also reported as a potential biomarker for colorectal cancer, detected in fecal DNA methylation profiles [27,31]. However, the expression pattern of *NDRG4* in esophageal adenocarcinoma has not been reported.

## 2. Results

### 2.1. Integrated Gene Expression Analysis Identified NDRG4 as One of the Top Candidates Silenced by DNA Methylation in EAC

**Figure 1:**
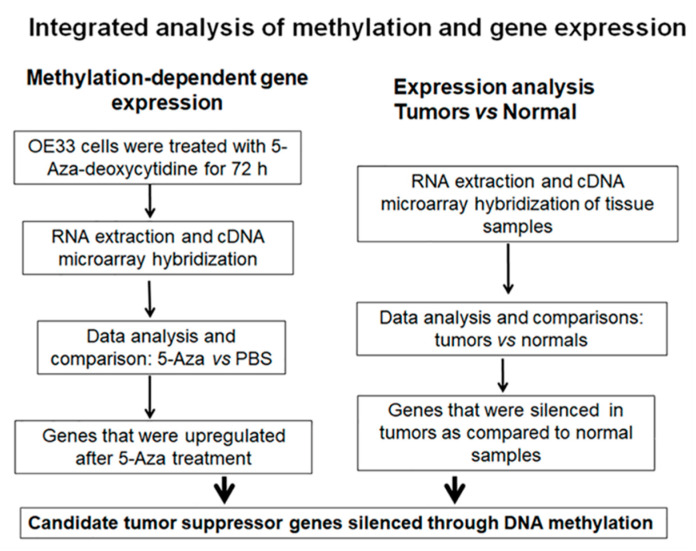
Experimental flow chart for screening functional epigenetic markers.

In an effort to identify potential tumor suppressor genes that are regulated through DNA methylation, we treated OE33 cells (an EAC cell line) with 5-aza-deoxycitidine (5-Aza) and performed cDNA microarray gene expression analysis. 5-Aza is a DNA methyltransferase inhibitor and has been used to restore gene expression silenced by DNA methylation [18]. At the same time, we carried out cDNA microarray analysis for 10 paired normal and EAC samples. The genes with significant upregulation after 5-Aza treatment (provided in Table S1) and genes with significant downregulation in EAC as compared to normal samples (provided in Table S2) were considered as most likely candidate genes methylated in EAC (the experimental flow chart is shown in Figure 1). We searched for CpG islands in the promoter regions and selected genes with a CpG island in their promoter region (CpG island finder, http://dbcat.cgm.ntu.edu.tw). The top 10 candidate genes are given in Table S3. Among these, we validated several genes using methylation specific PCR (MSP) technology, and selected *NDRG4* for further validation and investigation.

### 2.2. NDRG4 Gene Expression Is Significantly Downregulated in Barrett’s Esophagus and EACs

**Figure 2:**
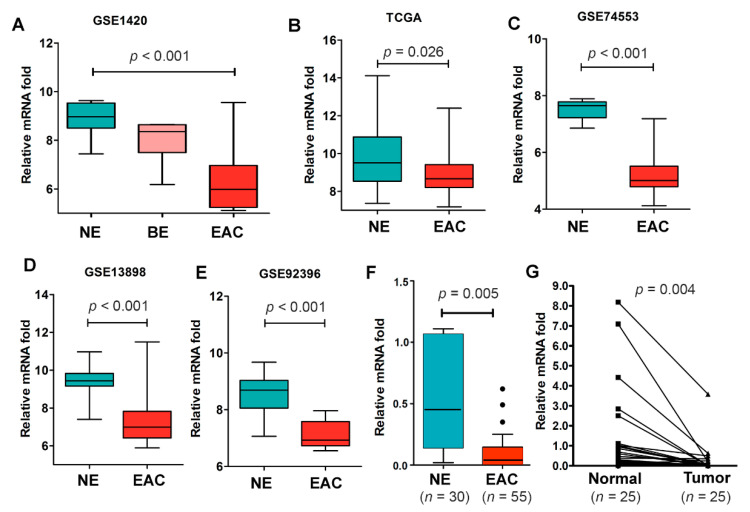
N-myc downregulated gene 4 (NDRG4) expression was frequently downregulated in Barrett’s esophagus and esophageal adenocarcinoma. (**A**–**E**) analysis of NDRG4 gene expression from available on-line data bases showed NDRG4 was downregulated in BE (**A**) and further downregulated in EAC. (**F**) qPCR analysis of NDRG4 gene expression in our own normal and tumor tissue samples (*p* = 0.005). (**G**) qPCR analysis of NDRG4 gene expression in 25 paired normal and tumor samples from the same patients confirmed downregulation of NDRG4 in EAC (*p* = 0.004). NE, normal esophagus; BE, Barrett’s esophagus; EAC, esophageal adenocarcinoma.

To confirm the above screening experiments’ results, we first searched the TCGA database and Gene Expression Omnibus (GEO) database for *NDRG4* gene expression in Barrett’s esophagus (BE) and EAC. We found that *NDRG4* was significantly downregulated in BE and further downregulated in EAC (GSE1420, Figure 2A), as compared to normal esophageal samples. Similar results were obtained from TCGA data (Figure 2B) and other data sets (Figure 2C–E). We validated the results using qPCR in primary EAC samples and normal esophageal samples (*p* = 0.005, Figure 2F), including 25 paired normal and tumor samples (*p* = 0.004, Figure 2G).

### 2.3. NDRG4 Promoter Is Hypermethylated in Barrett’s Esophagus and EACs

**Figure 3:**
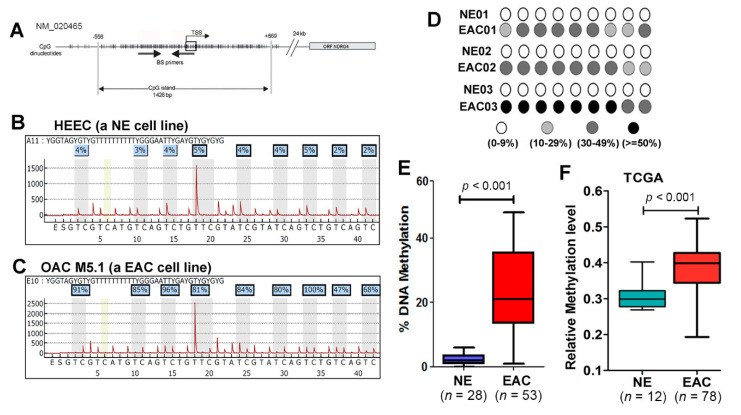
NDRG4 promoter CpG sites were frequently hypermethylated in esophageal adenocarcinoma. (**A**) a schematic drawing shows the structure of NDRG4 promoter region. There is a large CpG island spanning from −556 to +869 of transcription start site (TSS). BS primers are for amplification of bisulfate-modified DNA for pyrosequencing. (**B**,**C**) representative Pyrosequencing histograms for HEEC (a NE cell line, (**B**) and OAC M5.1 (an EAC cell line, (**C**) showing DNA methylation levels for each of the nine individual CpG sites assayed. (**D**) DNA methylation levels of NDRG4 promoter in three representative paired NE and EAC tissue samples. (**E**) analysis of average NDRG4 methylation levels from all NE and EAC samples examined using Pyrosequencing. Data shows significantly higher methylation levels in EAC than that in NE (*p* < 0.001). (**F**) analysis of NDRG4 methylation levels from TCGA data set confirmed significantly elevated DNA methylation levels in EAC than that in NE (*p* < 0.001).

**Figure 4:**
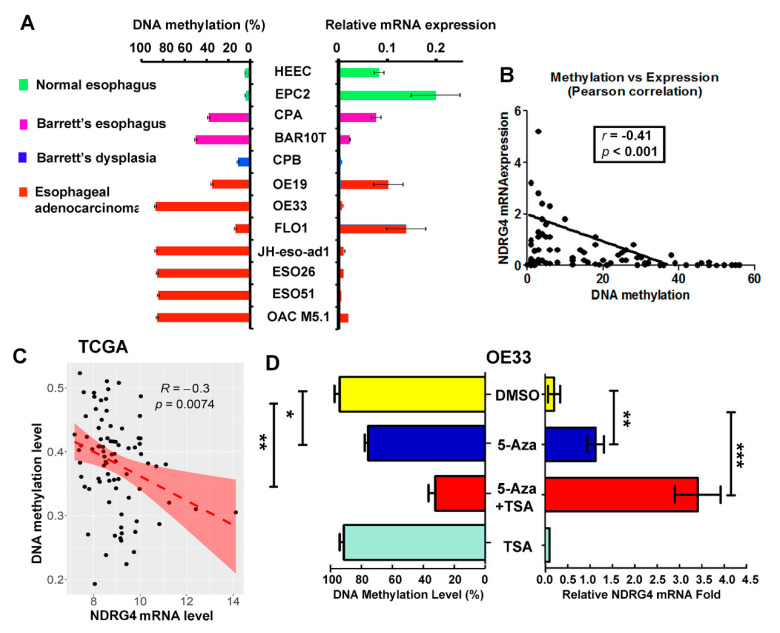
NDRG4 promoter DNA methylation levels inversely correlated with NDRG4 gene expression. (**A**) Side by side comparison of NDRG4 promoter DNA methylation levels detected by pyrosequencing (left) with NDRG4 gene expression levels by qPCR (right) in 12 esophageal cell lines. (**B**) Pearson correlation analysis of NDRG4 promoter DNA methylation levels with NDRG4 gene expression levels in all samples (*n* = 81) demonstrated an inverse correlation. (**C**) Correlation analysis of DNA methylation and gene expression data of NDRG4 from TCGA database (*n* = 81) confirmed an inverse relationship between NDRG4 DNA methylation and gene expression. (**D**) OE33 cells were treated with 5 µM 5-Aza alone or in combination with 100 nM TSA. qPCR was used to evaluate relative mRNA fold change of NDRG4 (right) and Pyrosequencing was used to quantitate NDRG4 methylation level change (left). * *p* < 0.05; ** *p* < 0.01; *** *p* < 0.001.

To find the potential mechanisms that underlie *NDRG4* gene downregulation, we first searched the Catalogue of Somatic Mutations in Cancer (COSMIC) data base (http://cancer.sanger.ac.uk/cosmic) for *NDRG4* somatic mutations. The *NDRG4* somatic mutations are very rare in EAC: only one of 286 EAC samples had a mutation. Because *NDRG4* methylation has been reported in colorectal cancers and our screening data showed downregulation of *NDRG4* expression that was reversed following 5-Aza treatment, we hypothesized that *NDRG4* promoter DNA hypermethylation may be the major mechanism to silence *NDRG4* in EAC. We searched the *NDRG4* promoter region and found a large CpG island around the transcription start site (TSS, Figure 3A). We first carried out methylation-specific PCR (MSP). MSP assay confirmed DNA methylation in EAC samples but not in normal esophagus (NE) (Figure S1). However, MSP could not quantitate DNA methylation level. Therefore, we applied a pyrosequencing assay that enabled us to quantitate DNA methylation for each individual CpG site. The representative pyrosequencing profiles in a normal esophagus cell line (HEEC) and an EAC cell line (OAC M5.1) are shown in Figure 3B,C, respectively. We, next, applied the pyrosequencing technology to our primary EAC samples. The DNA methylation profiles from three representative normal esophagus (NE) and matched EAC are shown in Figure 3D. The average DNA methylation level of NE samples tested were all below 10%, while DNA methylation level in EAC were significantly higher than that in NE (*p* < 0.001, Figure 3E). Analysis of DNA methylation data from TCGA database obtained similar results (*p* < 0.001, Figure 3F). We tested 12 esophageal cell lines; the two normal esophageal cell lines were unmethylated for *NDRG4*. However, the *NDRG4* promoter was partially methylated in two Barrett’s esophagus cell lines, CPA and BAR10T, and it was highly methylated in the majority of EAC cell lines (Figure 4A). 

### 2.4. NDRG4 Promoter Methylation Level Is Inversely Correlated with NDRG4 Gene Expression

To analyze the relationship between *NDRG4* promoter methylation and gene expression, we first compared the DNA methylation levels of the *NDRG4* promoter with the mRNA expression levels of the 12 esophageal cell lines side by side (Figure 4A). Generally, an inverse correlation was observed between DNA methylation levels and gene expression levels; lower or silence of *NDRG4* mRNA were seen in BE lines and EAC cell lines. Accordingly, we observed lower NDRG4 protein levels in BE and EAC cell lines (Figure S2). We then carried out a correlation analysis using Prism software and data from our primary samples (*n* = 81). The *NDRG4* DNA methylation level was significantly inversely correlated with *NDRG4* gene expression (*r* = −0.41, *p* < 0.001, Figure 4B). Analysis of TCGA data set for DNA methylation and gene expression (81 out of 90 samples available for both gene expression and DNA methylation) of *NDRG4* confirmed the inverse correlation (*r* = −0.3, *p* = 0.0074, Figure 4C). Furthermore, we treated two EAC cell lines (OE33 and JH-eso-ad1) in which *NDRG4* is significantly downregulated with 5-Aza and TSA (Trichostatin A, a histone deacetylase inhibitor). As expected, 5-Aza alone or in combination with TSA significantly restored *NDRG4* expression with demethylation of *NDRG4* promoter DNA in both OE33 (Figure 4D) and JH-eso-ad1 (Figure S3). However, TSA alone failed to obviously restore *NDRG4* expression.

### 2.5. Reconstitution of NDRG4 Expression Inhibited Tumor Cells Growth

**Figure 5:**
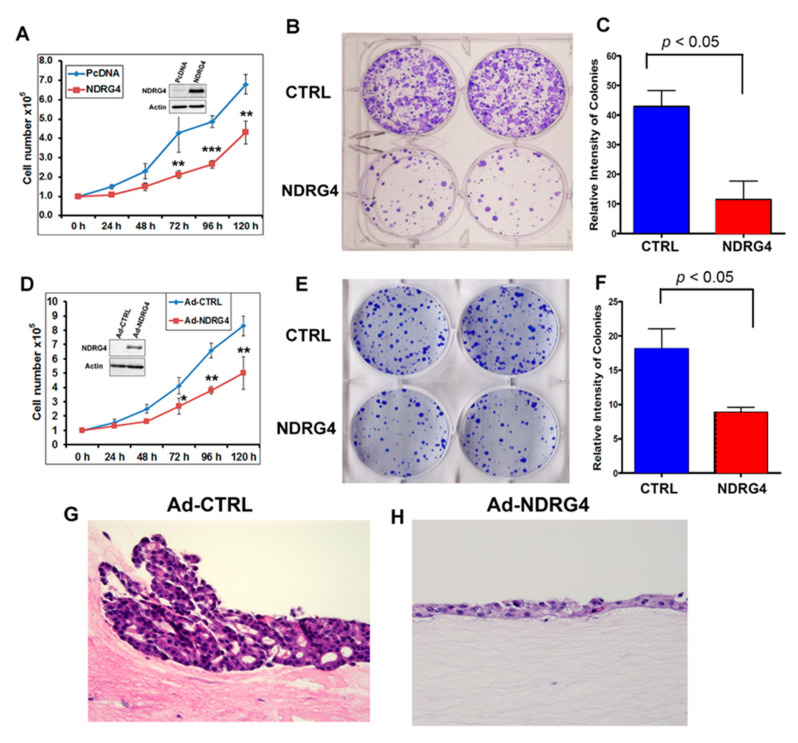
Exogenous expression of NDRG4 suppressed EAC cell growth. (**A**–**C**) FLO1 cells were forced expression of NDRG4 using PcDNA plasmid model followed by performing cell growth curve (**A**) and colony formation assay (**B**). (**C**) shows relative intensity of colonies quantitated using Image J. (**D**–**F**) JH-eso-ad1 cells were forced expression of NDRG4 using adenoviral model, followed by performing a cell growth curve (**D**) and colony formation assay (**E**). (**F**) shows the relative intensity of colonies quantitated using Image J. (**G**,**H**) OE19 cells were infected with ad-CTRL or ad-NDRG4 adenovirus particles (10 MOI), then applied to the three-dimensional (3D) organotypic culture model. Cell cultures were harvested after two weeks, fixed in 70% ethanol, and embedded in paraffin. HE staining is shown (magnification, 200×), (**G**) for ad-CTRL and (**H**) for ad-NDRG4.

To investigate the NDRG4 function, we reconstituted NDRG4 into two EAC cell lines, FLO1 and JH-eso-ad1. The reconstitution of NDRG4 significantly suppressed tumor cell growth as shown in Figure 5A,D, the cell growth curves (A for FLO1 and D for JH-eso-ad1). We performed a long-term colony formation assay after reconstitution of NDRG4. The colony formation assay confirmed that NDRG4 inhibited tumor cell colony formation capacity in FLO1 cells (Figure 5B,C, *p* < 0.05) and JH-eso-ad1 cells (Figure 5E,F, *p* < 0.05). To mimic the tumor cell growth pattern in vivo, we applied a three-dimensional (3D) organotypic culture (OTC) model. The OTC system allows for the co-culture of immortalized human epithelial cell lines together with primary fibroblasts in 3D tissue reconstructions; this represents a novel means by which to perform in vitro experiments that are still physiologically relevant [32]. The results further confirmed that reconstitution of NDRG4 suppressed tumor cell growth, as indicated by thinner cell layers in NDRG4 expressing cells as compared to control cells (Figure 5G,H).

### 2.6. NDRG4 Is Involved in Cell Cycle Regulation through Downregulating Cyclin D1, CDK4, and CDK6 Expression in EAC Cells

**Figure 6:**
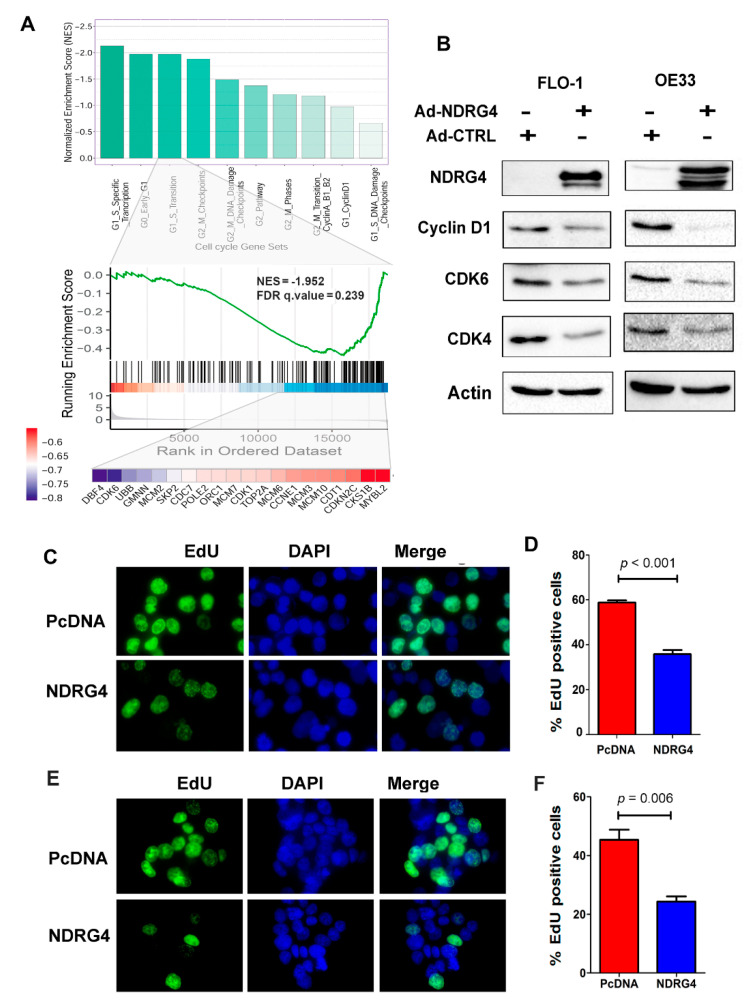
NDRG4 expression inhibited EAC cell proliferation through deregulation of cell cycle regulators. (**A**) Gene set enrichment analysis (GSEA) was performed in TCGA cohorts to assess the enrichment score of 10 cell cycle gene sets (derived from MSigDB). Normalization enrichment score (NES) values for these gene sets were presented in the bar graph (Up panel). G1/S transition regulators were significantly enriched in TCGA cohorts (Middle, NES = −1.952, FDR q value = 0.239). A total of the 20 most different expression genes, including CDK6, were shown in the heatmap (Lower panel). Values in the heatmap indicated log2 (fold change) between high and low expression of NDRG4. (**B**) western blotting analysis of cell cycle regulators; Cyclin D1, CDK4, CDK6 in FLO1 (left) and OE33 (right) cells with NDRG4 expression (Ad-NDRG4) and control cells (Ad-Ctrl). Detail information can be found at Figure S5. (**C**,**E**) representative images of EdU incorporation assay in FLO1 (**C**) and OE33 (**E**) cells with NDRG4 expression (NDRG4) and control cells (PcDNA). Green cells stand for EdU positive cells. DAPI (blue) was used to stain nucleus (magnification, 400×). (**D**) (FLO1) and (**F**) (OE33) show quantitation of EdU positive cells using Image J and statistically analyzed using Prism software.

To explore the potential underlying molecular mechanism of NDRG4-mediated cell growth suppression in EAC, we carried out a pathway analysis based on the TCGA database and a GEO database. The analysis identified that NDRG4 may be involved in some interesting pathways, such as a positive correlation with p53 pathway, apoptosis pathway, K-RAS downregulated pathway, and an inverse correlation with G2/M pathway and MYC pathway (Figure S4). Gene Set Enrichment Analysis (GSEA) indicated that genes involving cell cycle regulation were enriched, including G1/S and G2/M regulators (Figure 6A). To validate this, we carried out western blotting analysis for gene expression of major G1/S cell cycle regulators through reconstitution of NDRG4 in EAC cells. Reconstitution of NDRG4 in FLO1 and OE33 cells significantly downregulated protein levels of Cyclin D1, CDK4, and CDK6 (Figure 6B), as compared to control cells.

### 2.7. Reconstitution of NDRG4 Inhibited EAC Cell Proliferation 

The above results indicated that NDRG4 may regulate cell proliferation in EAC. To investigate if NDRG4 executes its tumor suppressor function in EAC through inhibiting tumor cell proliferation, we carried out an EdU (5-ethynyl-2′-deoxyuridine) cell proliferation assay in FLO1 and OE33 cell lines. An EdU assay was used as an alternate for the traditional BrdU incorporation assay to evaluate cell proliferation. Reconstitution of NDRG4 into tumor cells significantly reduced EdU positive rates in both FLO1 cells (*p* < 0.001, Figure 6C,D) and OE33 cells (*p* = 0.006, Figure 6E,F), suggesting a significant inhibition of cell proliferation. 

## 3. Discussion

Esophageal adenocarcinoma has become a major clinical challenge due to its continuing rapid increase of incidence in Western countries and poor overall prognosis [33,34]. Understanding the molecular events underlying the Barrett’s metaplasia-dysplasia-carcinoma sequence is a key step towards the development of early diagnosis and effective therapeutic strategies. In an effort to identify the potential molecular markers associated with DNA methylation, a major epigenetic regulation mechanism of gene expression, we performed an integrative functional epigenetic approach using 5-Aza (a DNA methyltransferase inhibitor) in combination with genome-wide analysis of gene expression of primary EAC samples. We identified a panel of genes that are likely regulated through the DNA methylation mechanism in EAC.

We focused on one of the top candidate genes, *NDRG4*, for further validation and investigation. We first confirmed that *NDRG4* gene expression was frequently downregulated in EAC tissue samples as compared to normal samples. We excluded somatic mutations and genomic variations as a driving force for downregulation of *NDRG4*. We demonstrated, using MSP and quantitative pyrosequencing technologies, that DNA hypermethylation of *NDRG4* promoter region is a frequent event in EAC. While the average methylation levels of the CpG sites was below 10% in normal esophageal samples, the majority of EAC samples tested showed more than 10% methylation levels. Moreover, we detected a significant inverse correlation between *NDRG4* methylation and expression levels. These data strongly suggest that *NDRG4* promoter DNA hypermethylation is a major mechanism in silencing NDRG4 in EAC. To further confirm this note, we treated two EAC cell lines with high DNA methylation levels and silence of *NDRG4* with 5-Aza, a DNA methyltransferase inhibitor. As expected, 5-Aza treatment or 5-Aza in combination with TSA significantly restored *NDRG4* expression. However, TSA alone failed to restore *NDRG4* expression. These results confirmed that *NDRG4* promoter hypermethylation, rather than histone acetylation, is the major force to downregulate NDRG4 in EAC. Interestingly, we observed that the combination of TSA administration following 5-Aza led to a bigger restoration of gene expression and DNA demethylation of NDRG4 in these cell lines. These results are in agreement with our previous observation [18] and other reports [35]. It has been reported that TSA could induce global and gene specific demethylation in human cancer cell lines [36,37]. TSA could decrease the mRNA stability of major DNA methyltransferases 3b (DNMT3b) [38] or activity of DNMT1 [35]. However, how these two agents interplay in this process is not clear and needs further investigation.

The role of NDRG4 in human cancers is quite controversial, in particular in tumors of the central nervous system [39]. In fact, NDRG4 is upregulated in tumors of central nerve system, such as glioblastoma [28] and meningioma [40], functioning as an oncogene. However, in gastrointestinal tract tumors, current evidences demonstrated that NDRG4 is downregulated in gastric cancers [26,29] and colorectal cancers [27,41], suggesting that it may function as a tumor suppressor. Our data, showing its downregulation in EAC, support the evidence that NDRG4 behaves differently in gastrointestinal tumors. Of note, the reconstitution of NDRG4 in EAC cells significantly inhibited tumor cell growth and cell proliferation, suggesting a tumor suppressor role in EAC.

The detection of DNA methylation as biomarkers of carcinogenesis has become an important topic due to its relative stability in tumor tissues, blood, stools, and body fluids [42,43,44]. Along these lines, *NDRG4* methylation has emerged as a useful DNA methylation marker, in combination with other markers, for colorectal cancers [27,45,46,47,48]. In the current study, we demonstrated that DNA hypermethylation was presented in 80% of EAC samples, but not in normal esophageal epithelial samples tested. Interestingly, two cell lines from Barrett’s esophagus demonstrated partial *NDRG4* methylation. It is known that BE is the precancerous condition for EAC. However, only a small proportion of patients with BE progress to dysplasia and finally EAC [1,49]. We suggest that *NDRG4* methylation may serve as a biomarker for EAC. However, to establish its utility as a biomarker, a large size patients’ cohort is needed. 

## 4. Methods and Materials 

### 4.1. Cell Lines

Detail information of cell lines used in the current study is provided in Table S4. All cell lines were grown at 37 °C in 5% carbon dioxide. We regularly authenticate the cell lines by sending them to Genetica Cell Line Test service utilizing STR DNA profiling technology. 

### 4.2. Tissue Samples

For DNA and mRNA analysis, 85 frozen tissue samples (55 EACs, 30 normal esophagus) were collected from the National Cancer Institute Cooperative Human Tissue Network (CHTN), including 25 paired normal and tumor samples from the same patients. The use of de-identified coded archival specimens was approved as non-human subject research by the Institutional Review Board. The normal esophagus samples were taken from tumor-free margins of resected tumors and were histologically confirmed normal without dysplastic changes. All EACs were anatomically originated from the lower esophagus or gastro-esophageal junction.

### 4.3. Integrated DNA Methylation-Dependent Gene Expression Analysis

To identify potential tumor suppressor genes that are regulated through DNA methylation, we treated OE33 cells (an EAC cell line) with 5-aza-deoxycitidine (5-Aza) at 5 µM for 72 h. RNA were extracted using Qiagen RNeasy mini kit and sent to Genomics Core Facility for cDNA microarray hybridization. We used Affymetrix Human Gene 1.0 ST arrays (Affymetrix, Santa Clara, CA, USA) for gene expression analysis. Differential expression analysis for primary samples was performed using limma package. The false discovery rate (FDR) method was used to adjust *p*-values for multiple comparisons. We picked up genes that were shown significant upregulation (with two-fold change) after 5-Aza treatment. At the same time, we performed cDNA microarray hybridization for gene expression from 10 primary EAC samples and their matched normal samples. In this case, we picked up genes that were silenced in EAC as compared to normal samples (with two-fold change and *p* < 0.05). Genes that were picked up in both data sets, upregulated by 5-Aza and silenced in EAC, were the candidate genes most likely silenced through DNA hypermethylation. A summary of experimental flow is shown in Figure 1.

### 4.4. Quantitative Real Time RT-PCR (qPCR)

Total RNA was purified using the RNeasy mini kit (Qiagen, Germantown, MD, USA). Single-stranded complementary DNA (cDNA) was synthesized from 1 µg RNA using the iScript cDNA synthesis Kit (Bio-Rad, Hercules, CA, USA). We chose the *HPRT* gene as a reference gene for all our qPCR normalization as used in our publications [18,50]. The primer sequences are provided in Table S5. The qPCR was performed in triplicate and the fold expression was calculated as previously reported [18,50,51].

### 4.5. DNA Bisulfite Modification and DNA Methylation Analyses

DNA was purified using the DNeasy Blood and Tissue Kit (Qiagen) and bisulfite modified using an EZ DNA Methylation Gold Kit (ZYMO Research, Orange, CA, USA) following the manufacturer’s protocol. We first performed methylation-specific PCR (MSP), which uses specific primers targeting the unmethylated DNA and methylated DNA in *NDRG4* promoter region, respectively. The primer sequences are provided in Table S6. To further quantitate DNA methylation levels of individual CpG sites in *NDRG4* promoter, we applied pyrosequencing technology. The pyrosequencing assay for *NDRG4* was purchased from Qiagen (Lot No. 201343589). The PCR conditions and subsequent pyrosequencing procedure followed the manufacturer’s protocol as in our previous reports [18,51]. For both MSP and Pyrosequencing assays, a universal methylated DNA (Zymo Research, Orange, CA, USA) was used as positive control at each run. 

### 4.6. 5-Aza-2′ Deoxycytidine (5-Aza) and Trichostatin A (TSA) Treatment

To validate the role of DNA methylation in *NDRG4* transcriptional regulation, two esophageal cancer cell lines (OE33 and JH-eso-ad1), in which *NDRG4* promoter is highly methylated and *NDRG4* gene expression is silenced, were treated with 5 μM 5-Aza (Sigma-Aldrich, St. Louis, MO, USA) for 72 h and/or 100 nM TSA (Wako, Osaka, Japan) for 24 h. Total RNA and DNA were isolated and purified using QIAGEN RNeasy mini kit and DNeasy Blood and tissue kit (QIAGEN). DNA methylation levels of NDRG4 were quantitated by pyrosequencing and NDRG4 mRNA expression levels were determined by qPCR as described above.

### 4.7. Cloning and Construction of NDRG4 Expression Plasmids

A full length of *NDRG4* coding sequence was amplified from normal cDNA by PCR using High Fidelity Platinum PCR SuperMix (Invitrogen, Carlsbad, CA, USA). PCR product was purified and cloned into the PcDNA 3.1 plasmid and pACCMV.pLpA plasmid. The pACCMV.pLpA-NDRG4 plasmid was co-transfected with pJM17 plasmid into 293 AD cells to generate and propagate the full adenoviral NDRG4 particles as previous described [50]. The viruses were plaque purified, and the titer of the virus was determined using the Adeno-X qPCR Titration Kit (Clontech, Mountain View, CA, USA). Esophageal adenocarcinoma cell lines, FLO-1, JH-eso-ad1 and OE33 were transfected with PcDNA control or NDRG4 plasmids using lipofectamine 2000 transfection reagent (ThermoFisher Scientific, Waltham, MA, USA), or were infected with 10 multiplicity of infection (MOI) per cell of adenoviral NDRG4 particles (Ad-NDRG4) and adenoviral empty particles (Ad-CTRL) in the culture medium. Forty-eight hours after transfection/infection, the cells were harvested for NDRG4 expression validation in both mRNA level using qPCR and in protein level by western blotting.

### 4.8. Determination of Cell Growth Curve

FLO-1 and JH-eso-ad1 cells were infected with 10 MOI control or NDRG4 expression adenoviral particles or transfected with PcDNA control or NDRG4 expression plasmids, followed by selection under 600 mg/mL G418 for two weeks. In total, 5 × 10^4^ cells per well in the 6-well plates were seeded and the cell numbers were counted at 24 h interval using trypan blue exclusion assay and Bio-rad TC20 Cell Counter (Bio-Rad). The experiments were repeated in triplicates and each sample was counted three times. Then, the readings were averaged for each sample.

### 4.9. Colony Formation Assay

FLO-1 and JH-eso-ad1 cells were infected with 10 MOI control or NDRG4 expression adenoviral particles. 48 h after infection, cells were split and seeded in the density of 1000 cells/well in the 6-well plates. Cells were cultured in full medium at 37 °C for another 2 weeks. Cells then were fixed and stained with 0.5% crystal violet solution. The number and intensity of colonies were analyzed using Image J software. Each experiment was set in triplicate and statistical analysis was performed using Prism software. 

### 4.10. EdU Cell Proliferation Assay

To measure cell proliferation, the Click-iT EdU Assay (Invitrogen) was used as previously described [52]. In brief, FLO1 and OE33 cells were transfected with PcDNA control and NDRG4 expression plasmids for 48 h. Equal volume of 2 × EdU solutions was added to the cells, and incubated at 37 °C for 60 min. After incubation, cells were fixed with 3.7% formaldehyde in PBS (Phosphate Buffered Saline) for 15 min. Permeabilization using 0.5% Triton X-100 for 20 min was followed. Then, Click-iT reaction cocktail was added to the cell culture medium and incubated for 30 min in dark. Click-iT reaction cocktail was then removed, followed by washing twice with 3% BSA (Bovine Serum Albumin) in PBS. Cell nucleus was stained with Vectashield antifade mounting medium with 4′,6-diamidino-2-phenylindole (DAPI) (Vector Laboratories, Inc., Burlinggame, CA, USA). At least 20 random fields at 20×, > 400 cells, were counted using Image J software. The percentage of EdU-positive cells versus total number of nucleus (DAPI stained) was calculated and statistically analyzed using Prism software.

### 4.11. Western Blotting Analysis

Cells were infected with Ad-NDRG4 or control virus (10 MOI) for 72 h. Then, cells were collected and lysed using RIPA (Radioimmunoprecipitation assay) buffer with supplements of proteinase inhibitors and phosphatase inhibitors (Santa Cruz, CA, USA). Western blotting was carried out following standard protocol. The antibody against NDRG4 was purchased from LSBio (LS-C133806, mouse monoclonal, Seattle, WA, USA). Antibodies against CyclinD1 (E3P5S, monoclonal), CDK4 (D9G3E, monoclonal), and CDK6 (DCS83, monoclonal) were purchased from Cell Signaling (Danvers, MA, USA). β-Actin (AC-74, Millipore Sigma, Miamisburg, OH, USA) was used as internal loading control of each experiment.

### 4.12. 3D Organotypic Cell Culture

3D organotypic culture (OTC) was performed as previously described [53]. In brief, first, human esophageal fibroblast (hEF) cells were seeded with the collagen/Matrigel matrices and culture at 37 °C in full medium for 1 week. At 7th day, 0.5 × 10^6^ of Ad-Ctrl and Ad-NDRG4 OE19 cells (24 h after infection) were seeded on the top of collagen/Matrigel matrices containing fibroblasts and grown in epithelial media for another 7 days. The cultures were harvested and fixed in 70% ethanol and embedded in paraffin for HE staining, histology evaluation and subsequent investigations. 

### 4.13. Gene Expression Databases

mRNA expression data of esophageal adenocarcinoma was downloaded from The Cancer Genome Atlas (TCGA) (https://portal.gdc.cancer.gov/) and Gene Expression Omnibus (GEO) (https://www.ncbi.nlm.nih.gov/gds) cohorts. The TCGA data includes 78 esophageal adenocarcinoma and nine normal esophageal epithelial samples. The GEO cohorts include GSE13898 (EAC: 75, NE: 28), GSE92396 (EAC: 12, NE: 10), GSE74553 (EAC: 52, NE: 8), GSE26886 (EAC: 20, NE: 17), and GSE1420 (EAC: 8, NE: 8). Gene annotation was performed using R language (R 3.6.1; https://www.r-project.org/). When a gene had multiple probes, the mean value of these probes was used to represent the gene expression level. Differential expression gene (DEG) analysis was performed with limma package.

### 4.14. Gene Set Enrichment Analysis

We selected EAC patients from TCGA and GSE74553 cohorts for gene set enrichment analysis (GSEA). The Mean ± SD of *NDRG4* expression levels were used as the cut-off points to divide the samples into three groups: high expression group (*n* = 19), median expression group (*n* = 90), and low expression group (*n* = 21). The DEGs were derived from high expression group compared to low expression group (the DEGs from TCGA and GSE74553 was provided in Tables S7 and S8, respectively), and clusterProfiler v3.12.0 package (https://guangchuangyu.github.io/software/clusterProfiler) was used for GSEA analysis. All hallmark gene sets and cell cycle gene sets were downloaded from MSigDB (https://www.gsea-msigdb.org/).

### 4.15. Statistical Analysis

All statistical analyses were done using GraphPad Prism 8 software. The differences between NE and EAC were compared using student’s *t* test. Pearson correlation was used to analyze the correlation between DNA methylation levels and gene expression levels. For all analyses, *p* < 0.05 was considered as significant. 

## 5. Conclusions

In summary, we have demonstrated that the *NDRG4* gene is frequently downregulated in esophageal adenocarcinoma through promoter DNA hypermethylation and may play a tumor suppressor role by inhibiting tumor cell proliferation.

## Supplementary Materials

The following are available online at https://www.mdpi.com/2072-6694/12/9/2573/s1, Figure S1: MSP for NDRG4, Figure S2: NDRG4 protein levels were downregulated in BE and EAC cell lines, Figure S3: 5-Aza restored NDRG4 gene expression in JH-eso-ad1 cells, Figure S4: Pathway analysis of NDRG4-related genes, Figure S5: Detail information about Figure 6B, Table S1: Upregulated genes after 5-Aza treatment in OE33, Table S2: Downregulated genes in EAC, Table S3: TOP 10 candidate genes, Table S4: Cell lines used in this study, Table S5: Sequences for qPCR primers, Table S6: Sequences for NDRG4 MSP primers, Table S7: Differential expression gene from TCGA used in Figure 6A, Table S8: Differential expression gene from GSE 74553 used in Figure 6A.

## References

[1] Altorki N.K., Skinner D.B. (1990). Adenocarcinoma in Barrett’s esophagus. Semin. Surg. Oncol..

[2] DeVault K.R. (2000). Epidemiology and significance of Barrett’s esophagus. Dig. Dis..

[3] Caygill C.P., Reed P.I., Johnston B.J., Hill M.J., Ali M.H., Levi S. (1999). A single centre’s 20 years’ experience of columnar-lined (Barrett’s) oesophagus diagnosis. Eur. J. Gastroenterol. Hepatol..

[4] Pera M. (2003). Trends in incidence and prevalence of specialized intestinal metaplasia, barrett’s esophagus, and adenocarcinoma of the gastroesophageal junction. World J. Surg..

[5] Falk G.W. (1994). Barrett’s esophagus. Gastrointest. Endosc. Clin. N. Am..

[6] Hoff S.J., Sawyers J.L., Blanke C.D., Choy H., Stewart J.R. (1998). Prognosis of adenocarcinoma arising in Barrett’s esophagus. Ann. Thorac. Surg..

[7] Falk G.W. (2001). Gastroesophageal reflux disease and Barrett’s esophagus. Endoscopy.

[8] Greenberg M.V.C., Bourc’his D. (2019). The diverse roles of DNA methylation in mammalian development and disease. Nat. Rev. Mol. Cell Biol..

[9] Jones P.A. (2012). Functions of DNA methylation: Islands, start sites, gene bodies and beyond. Nat. Rev. Genet..

[10] Edwards J.R., Yarychkivska O., Boulard M., Bestor T.H. (2017). DNA methylation and DNA methyltransferases. Epigenetics Chromatin.

[11] Pfeifer G.P. (2018). Defining Driver DNA methylation changes in human cancer. Int. J. Mol. Sci..

[12] Auerkari E.I. (2006). Methylation of tumor suppressor genes p16(INK4a), p27(Kip1) and E-cadherin in carcinogenesis. Oral. Oncol..

[13] Kulis M., Esteller M. (2010). DNA methylation and cancer. Adv. Genet..

[14] Kaz A.M., Grady W.M., Stachler M.D., Bass A.J. (2015). Genetic and Epigenetic Alterations in Barrett’s Esophagus and Esophageal Adenocarcinoma. Gastroenterol. Clin. N. Am..

[15] Hardie L.J., Darnton S.J., Wallis Y.L., Chauhan A., Hainaut P., Wild C.P., Casson A.G. (2005). p16 expression in Barrett’s esophagus and esophageal adenocarcinoma: Association with genetic and epigenetic alterations. Cancer Lett..

[16] Agarwal A., Polineni R., Hussein Z., Vigoda I., Bhagat T.D., Bhattacharyya S., Maitra A., Verma A. (2012). Role of epigenetic alterations in the pathogenesis of Barrett’s esophagus and esophageal adenocarcinoma. Int. J. Clin. Exp. Pathol..

[17] Peng D., Hu T.L., Jiang A., Washington M.K., Moskaluk C.A., Schneider-Stock R., El-Rifai W. (2011). Location-specific epigenetic regulation of the metallothionein 3 gene in esophageal adenocarcinomas. PLoS ONE.

[18] Peng D.F., Razvi M., Chen H., Washington K., Roessner A., Schneider-Stock R., El-Rifai W. (2009). DNA hypermethylation regulates the expression of members of the Mu-class glutathione S-transferases and glutathione peroxidases in Barrett’s adenocarcinoma. Gut.

[19] Fitzgerald R.C. (2009). Epigenetic changes wipe out protective mechanisms in Barrett’s oesophagus. Gut.

[20] Melotte V., Qu X., Ongenaert M., van Criekinge W., de Bruine A.P., Baldwin H.S., van Engeland M. (2010). The N-myc downstream regulated gene (NDRG) family: Diverse functions, multiple applications. FASEB J..

[21] Zhang J., Chen S., Zhang W., Liu X., Shi H., Che H., Wang W., Li F., Yao L. (2008). Human differentiation-related gene NDRG1 is a Myc downstream-regulated gene that is repressed by Myc on the core promoter region. Gene.

[22] Lorentzen A., Vogel L.K., Lewinsky R.H., Saebo M., Skjelbred C.F., Godiksen S., Hoff G., Tveit K.M., Lothe I.M., Ikdahl T. (2007). Expression of NDRG2 is down-regulated in high-risk adenomas and colorectal carcinoma. BMC Cancer.

[23] Shi H., Li N., Li S., Chen C., Wang W., Xu C., Zhang J., Jin H., Zhang H., Zhao H. (2010). Expression of NDRG2 in esophageal squamous cell carcinoma. Cancer Sci..

[24] Lorentzen A., Lewinsky R.H., Bornholdt J., Vogel L.K., Mitchelmore C. (2011). Expression profile of the N-myc Downstream Regulated Gene 2 (NDRG2) in human cancers with focus on breast cancer. BMC Cancer.

[25] Kim Y.J., Yoon S.Y., Kim J.T., Song E.Y., Lee H.G., Son H.J., Kim S.Y., Cho D., Choi I., Kim J.H. (2009). NDRG2 expression decreases with tumor stages and regulates TCF/beta-catenin signaling in human colon carcinoma. Carcinogenesis.

[26] Chen X., Yang Y., Liu J., Li B., Xu Y., Li C., Xu Q., Liu G., Chen Y., Ying J. (2017). NDRG4 hypermethylation is a potential biomarker for diagnosis and prognosis of gastric cancer in Chinese population. Oncotarget.

[27] Melotte V., Lentjes M.H., van den Bosch S.M., Hellebrekers D.M., de Hoon J.P., Wouters K.A., Daenen K.L., Partouns-Hendriks I.E., Stessels F., Louwagie J. (2009). N-Myc downstream-regulated gene 4 (NDRG4): A candidate tumor suppressor gene and potential biomarker for colorectal cancer. J. Natl. Cancer Inst..

[28] Schilling S.H., Hjelmeland A.B., Radiloff D.R., Liu I.M., Wakeman T.P., Fielhauer J.R., Foster E.H., Lathia J.D., Rich J.N., Wang X.F. (2009). NDRG4 is required for cell cycle progression and survival in glioblastoma cells. J. Biol. Chem..

[29] Zhang Z., She J., Yang J., Bu X., Ji G., Zhu S., He S., Chu D. (2018). NDRG4 in gastric cancer determines tumor cell proliferation and clinical outcome. Mol. Carcinog..

[30] Vaes N., Schonkeren S.L., Brosens E., Koch A., McCann C.J., Thapar N., Hofstra R.M.W., van Engeland M., Melotte V. (2018). A combined literature and in silico analysis enlightens the role of the NDRG family in the gut. Biochim. Biophys. Acta Gen. Subj..

[31] Iannone A., Losurdo G., Pricci M., Girardi B., Massaro A., Principi M., Barone M., Ierardi E., Di Leo A. (2016). Stool Investigations for Colorectal Cancer Screening: From Occult Blood Test to DNA Analysis. J. Gastrointest. Cancer.

[32] Nakagawa H., Whelan K., Lynch J.P. (2015). Mechanisms of Barrett’s oesophagus: Intestinal differentiation, stem cells, and tissue models. Best Pract. Res. Clin. Gastroenterol..

[33] Thrift A.P. (2018). Barrett’s Esophagus and Esophageal Adenocarcinoma: How Common Are They Really?. Dig. Dis. Sci..

[34] El-Serag H.B. (2002). The epidemic of esophageal adenocarcinoma. Gastroenterol. Clin. N. Am..

[35] Yang X., Phillips D.L., Ferguson A.T., Nelson W.G., Herman J.G., Davidson N.E. (2001). Synergistic activation of functional estrogen receptor (ER)-alpha by DNA methyltransferase and histone deacetylase inhibition in human ER-alpha-negative breast cancer cells. Cancer Res..

[36] Ou J.N., Torrisani J., Unterberger A., Provencal N., Shikimi K., Karimi M., Ekstrom T.J., Szyf M. (2007). Histone deacetylase inhibitor Trichostatin A induces global and gene-specific DNA demethylation in human cancer cell lines. Biochem. Pharmacol..

[37] Wu L.P., Wang X., Li L., Zhao Y., Lu S., Yu Y., Zhou W., Liu X., Yang J., Zheng Z. (2008). Histone deacetylase inhibitor depsipeptide activates silenced genes through decreasing both CpG and H3K9 methylation on the promoter. Mol. Cell Biol..

[38] Xiong Y., Dowdy S.C., Podratz K.C., Jin F., Attewell J.R., Eberhardt N.L., Jiang S.W. (2005). Histone deacetylase inhibitors decrease DNA methyltransferase-3B messenger RNA stability and down-regulate de novo DNA methyltransferase activity in human endometrial cells. Cancer Res..

[39] Schonkeren S.L., Massen M., van der Horst R., Koch A., Vaes N., Melotte V. (2019). Nervous NDRGs: The N-myc downstream-regulated gene family in the central and peripheral nervous system. Neurogenetics.

[40] Kotipatruni R.P., Ren X., Thotala D., Jaboin J.J. (2015). NDRG4 is a novel oncogenic protein and p53 associated regulator of apoptosis in malignant meningioma cells. Oncotarget.

[41] Chu D., Zhang Z., Zhou Y., Li Y., Zhu S., Zhang J., Zhao Q., Ji G., Wang W., Zheng J. (2015). NDRG4, a novel candidate tumor suppressor, is a predictor of overall survival of colorectal cancer patients. Oncotarget.

[42] Pan Y., Liu G., Zhou F., Su B., Li Y. (2018). DNA methylation profiles in cancer diagnosis and therapeutics. Clin. Exp. Med..

[43] Locke W.J., Guanzon D., Ma C., Liew Y.J., Duesing K.R., Fung K.Y.C., Ross J.P. (2019). DNA Methylation Cancer Biomarkers: Translation to the Clinic. Front. Genet..

[44] Tang Q., Cheng J., Cao X., Surowy H., Burwinkel B. (2016). Blood-based DNA methylation as biomarker for breast cancer: A systematic review. Clin. Epigenetics.

[45] Lam K., Pan K., Linnekamp J.F., Medema J.P., Kandimalla R. (2016). DNA methylation based biomarkers in colorectal cancer: A systematic review. Biochim. Biophys. Acta.

[46] Dong L., Ren H. (2018). Blood-based DNA Methylation Biomarkers for Early Detection of Colorectal Cancer. J. Proteom. Bioinform..

[47] Chen J., Sun H., Tang W., Zhou L., Xie X., Qu Z., Chen M., Wang S., Yang T., Dai Y. (2019). DNA methylation biomarkers in stool for early screening of colorectal cancer. J. Cancer.

[48] Lu H., Huang S., Zhang X., Wang D., Zhang X., Yuan X., Zhang Q., Huang Z. (2014). DNA methylation analysis of SFRP2, GATA4/5, NDRG4 and VIM for the detection of colorectal cancer in fecal DNA. Oncol. Lett..

[49] Dong J., Buas M.F., Gharahkhani P., Kendall B.J., Onstad L., Zhao S., Anderson L.A., Wu A.H., Ye W., Bird N.C. (2018). Determining Risk of Barrett’s Esophagus and Esophageal Adenocarcinoma Based on Epidemiologic Factors and Genetic Variants. Gastroenterology.

[50] Peng D., Belkhiri A., Hu T., Chaturvedi R., Asim M., Wilson K.T., Zaika A., El-Rifai W. (2011). Glutathione peroxidase 7 protects against oxidative DNA damage in oesophageal cells. Gut.

[51] Peng D., Hu T., Soutto M., Belkhiri A., Zaika A., El-Rifai W. (2013). Glutathione peroxidase 7 has potential tumour suppressor functions that are silenced by location-specific methylation in oesophageal adenocarcinoma. Gut.

[52] Chen Z., Hu T., Zhu S., Mukaisho K., El-Rifai W., Peng D.F. (2017). Glutathione peroxidase 7 suppresses cancer cell growth and is hypermethylated in gastric cancer. Oncotarget.

[53] Kalabis J., Wong G.S., Vega M.E., Natsuizaka M., Robertson E.S., Herlyn M., Nakagawa H., Rustgi A.K. (2012). Isolation and characterization of mouse and human esophageal epithelial cells in 3D organotypic culture. Nat. Protoc..

