# Supplementary material for: N-MYC Downstream Regulated Gene 4 (NDRG4), a Frequent Downregulated Gene through DNA Hypermethylation, plays a Tumor Suppressive Role in Esophageal Adenocarcinoma
Source: Cancers (Basel). 2020 Sep 10;12(9):2573. doi: 10.3390/cancers12092573 (PMC7565689; doi:10.3390/cancers12092573)
Supplement: Supplementary file 1 [file cancers-12-02573-s001.zip › cancers-909968-supply/Cancers-909968- supplementary .pdf]

Supplementary Materials

N-MYC Downstream Regulated Gene 4 (*NDRG4*), a Frequent Downregulated Gene through DNA Hypermethylation, plays a Tumor Suppressive Role in Esophageal Adenocarcinoma

Longlong Cao, Tianling Hu, Heng Lu and Dunfa Peng

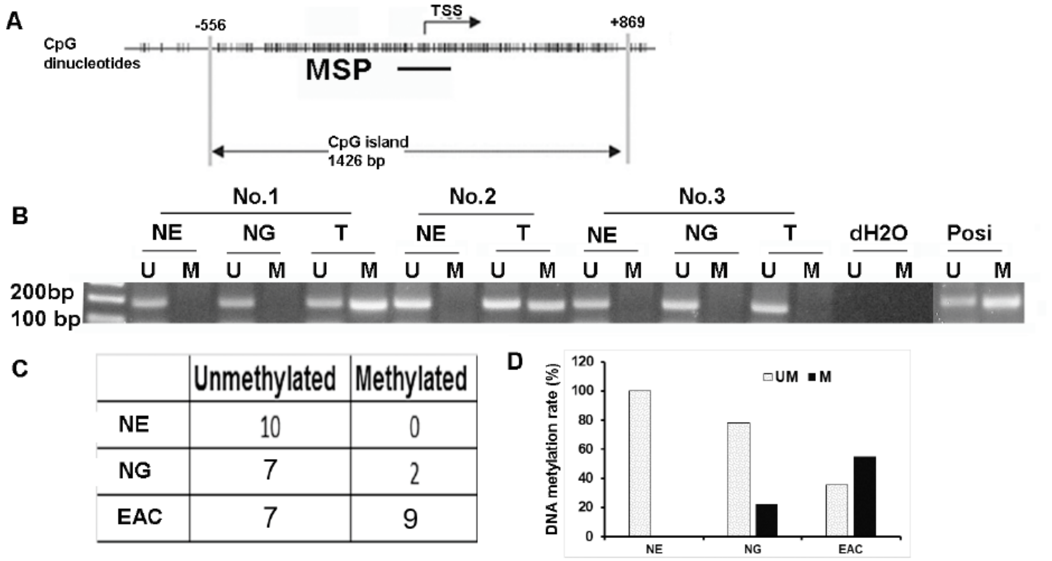

Figure S1. Methylation-specific PCR for NDRG4. (**A**) a schematic drawing shows the structure of NDRG4 promoter region. There is a large CpG island spanning from -556 to + 869 of transcription start site (TSS). A MSP primer set (including 4 primers) was designed to cover TSS. (**B**) Representative MSP images from 3 primary normal and tumor samples. U stands for unmethylated bands, M stands for methylated bands. Posi means positive controls for unmethylated DNA and methylated DNA. NE, normal esophagus; NG, normal gastric mucosa. (**C**) and (**D**) summary of NDRG4 methylation states in samples examined.

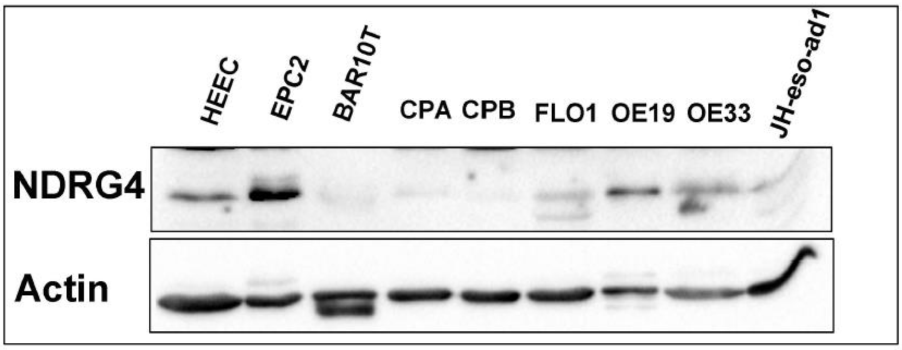

Figure S2. NDRG4 protein levels were downregulated in BE and EAC cell lines. Western blotting analysis of NDRG4 protein in esophageal cell lines. NDRG4 protein levels were higher in two normal esophagus cell lines; HEEC and EPC2, but down regulated in BE and majority of EAC cell lines.

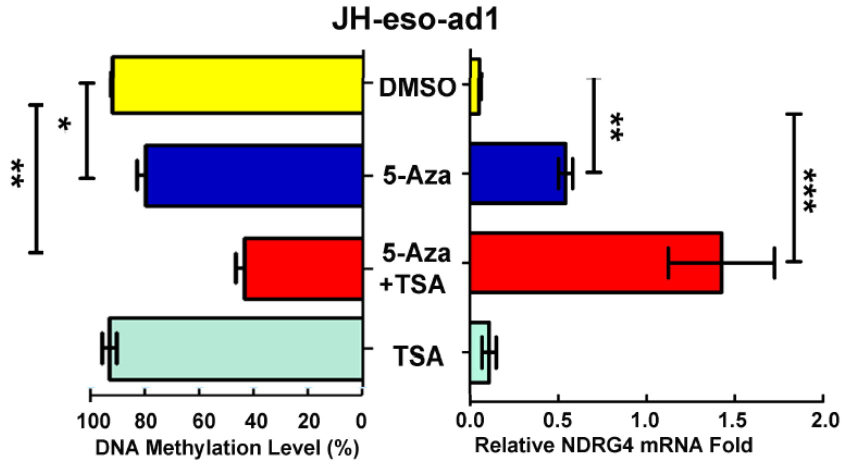

Figure S3. 5-Aza restored NDRG4 gene expression in JH-eso-ad1 cells. Tumor cells were treated with 5 μM 5-Aza and 100 nM TSA alone or in combination. qPCR was used to evaluate relative mRNA fold change of NDRG4 (right side) and Pyrosequencing was applied to quantitate NDRG4 methylation level change (left side). $^{*}p < 0.05$; $^{**}p < 0.01$; $^{***}p < 0.001$.

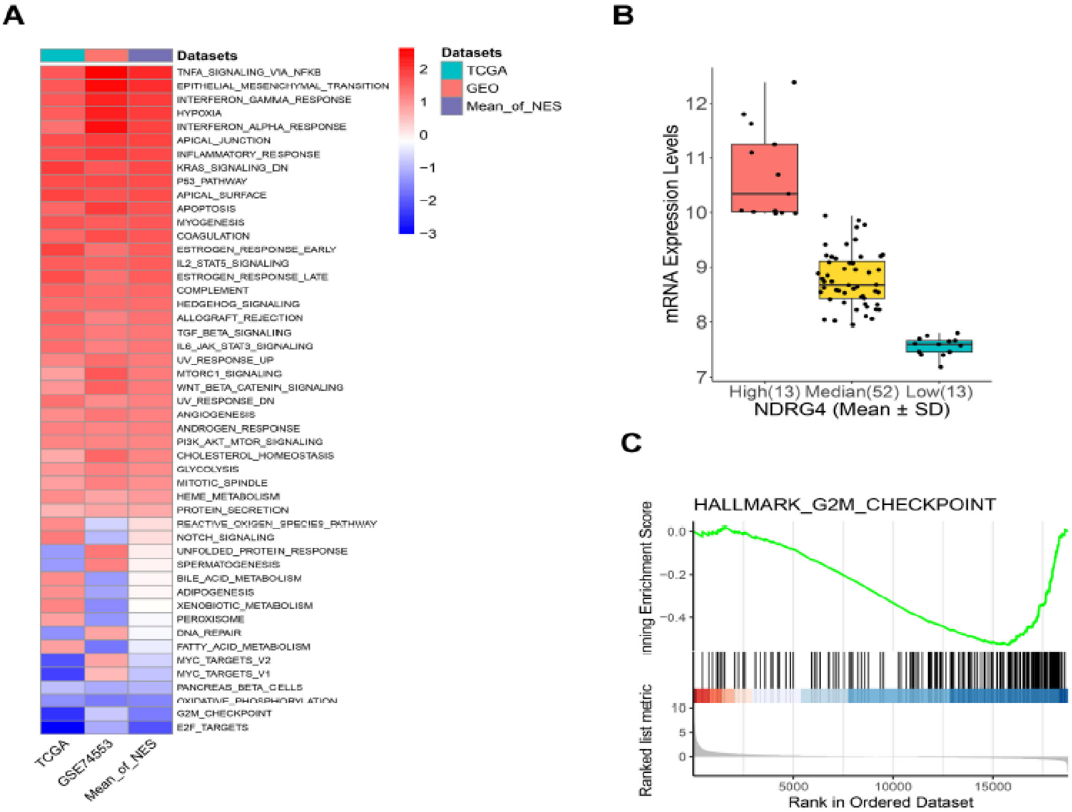

Figure S4. Pathway analysis of NDRG4-related genes. (**A**) a heatmap showed Normalization Enrichment score (NES) values of 50 hallmark gene sets (archived from MSigDB) in TCGA. cohorts and GSE74553 dataset by GSEA analysis. (**B**) The Mean ± SD of NDRG4 expression levels were used as the cut-off points to separate the TCGA samples into three groups: high group (n = 13), median group (n = 52), and low group (n = 13). (**C**) G2/M pathway can be obviously enriched in the NDRG4 low expression group in TCGA cohort (NES = -2.507, FDR q value = 0.085).

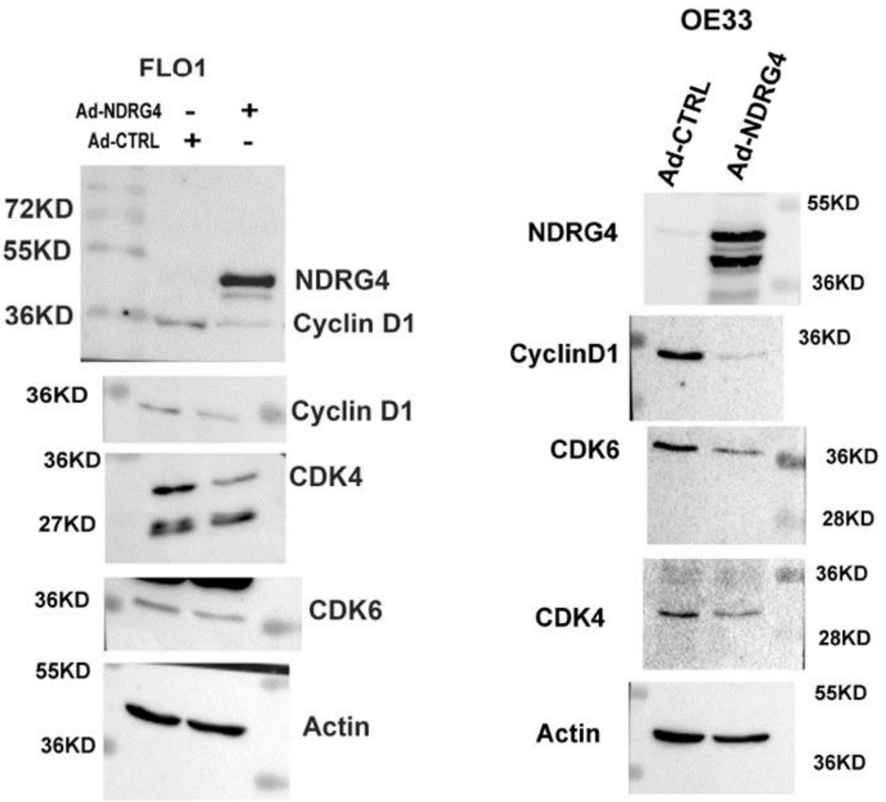

Figure S5. Detail information about Figure 6B.

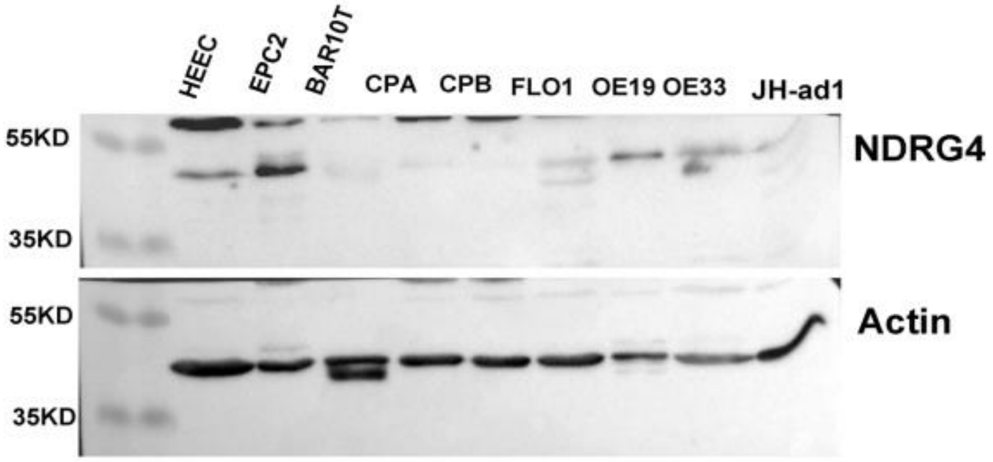

Detail information about Figure S2.

Table S3. TOP candidate genes.

Symbol	CpG Island	Validated by MSP		
			Normal	EAC
**MT3**	Yes	yes	15.8% (3/19)	68.8% (11/16)
**NDRG4**	yes	yes	10.5% (2/19)	56.3% (9/16)
**G0S2**	yes	yes	52.6% (10/19)	56.3% (9/16)
**MLLT7 (FOXO4)**	yes	yes	21.1% (4/19)	18.8% (3/16)
**INA**	yes	yes	15.8% (3/19)	75.0% (12/16)
**GAGE**	yes	yes	100% (19/19)	100% (16/16)
**PLCD1**	yes	yes	10.5% (2/19)	12.5% (2/16)
**MT1H**	yes	NA	NA	NA
**NDRG1**	yes	NA	NA	NA
**CA2**	yes	NA	NA	NA

Table S4. Cell lines used in this study.

	Name	Sources	Medium	FBS
Normal esophagus	HEEC	ScienCell Research Laboratories, CA, USA	EpiCM-2	5%
	EPC2	Dr. Hiroshi Nakagawa	EpiCM-2	5%
Barrett's esophagus	BAR-10T	Dr. Rhonda Souza	EpiCM-2	5%
	CPA	American Type Culture Collection, ATCC	EpiCM-2	5%
Dysplasia	CPB	ATCC	EpiCM-2	5%
Esophageal adeno-carcinoma	FLO1	ATCC	DMEM	10%
	OE19	Sigma-Aldrich, St Louis, Missouri, USA	RPMI 1640	10%
	ESO26	Sigma-Aldrich, St Louis, Missouri, USA	DMEM	10%
	ESO51	Sigma-Aldrich, St Louis, Missouri, USA	DMEM	10%
	OACM5.1	Sigma-Aldrich, St Louis, Missouri, USA	DMEM	10%
	OE33	Drs David Beer and Jim Eshleman	RPMI 1640	10%
	JH-eso-Ad1	Drs David Beer and Jim Eshleman	DMEM	10%
Esophageal fibroblast	hEF	ScienCell Research Laboratories, CA, USA	DMEM	10%
Kidney	HEK293 AD	Cell BioLabs Inc, San Diego, CA, USA	DMEM	10%

Table S5. Sequences for qPCR primers.

**The Sequence of *NDRG4* Primers for qPCR are:**
Forward, 5′-GGCCTTCTGCATGTAGTGATCCG-3′
Reverse, 5′-GGTGATCTCCTGCATGTCCTCG-3′.
**The Sequence of *HPRT* Primers for qPCR are:**
Forward, 5′- TTGGAAAGGGTGTTT ATTCCTCA -3′
Reverse, 5′- TCCAGCAGGTCAGCAAAGAA-3′.

Table S6. Sequences for *NDRG4* MSP primers.

**The Primer Sets for Unmethylated DNA are:**
Forward, 5′-GGA TTA GTT TTA GGT TTG GTA TTG TTT TGT-3′
Reverse, 5′-ACA AAA CTT CAA CAC CAC CAA CAC ACA-3′.
**The Primer Sets for Methylated DNA are:**
Forward, 5′-TTA GTT TTA GGT TCG GTA TCG TTT CGC-3′
Reverse, 5′-CTT CGA CGC CGC CGA CGC GCG-3′.

© 2020 by the authors. Licensee MDPI, Basel, Switzerland. This article is an open access article distributed under the terms and conditions of the Creative Commons Attribution (CC BY) license (http://creativecommons.org/licenses/by/4.0/).
